# Real-life use of fluticasone propionate/salmeterol in patients with chronic obstructive pulmonary disease: a French observational study

**DOI:** 10.1186/1471-2466-14-56

**Published:** 2014-04-02

**Authors:** Nicolas Roche, Céline Pribil, Eric Van Ganse, Philippe Serrier, Bruno Housset, Déborah Poirier, Nathalie Texier, Stéphane Schück, Isabelle Boucot

**Affiliations:** 1 Respiratory and Intensive Care Medicine, Cochin Hospital Group, AP-HP, University Paris Descartes, HIA du Val de Grâce 4e C, Paris, France; 2 Department of Pharmacoepidemiology and Medico-Economic Modelling, GlaxoSmithKline, Marly Le Roi, France; 3 Department of Pharmacoepidemiology, CHU-Lyon, Lyon, France; 4 Private Medical Practice, Paris, France; 5 Department of Pulmonology, CHI Creteil Hospital, Créteil, France; 6 Kappa Santé, Paris, France; 7 Respiratory and Immuno-Inflammation Department, GlaxoSmithKline, Marly Le Roi, France

**Keywords:** COPD, France, GOLD, Prescription, Questionnaire, Population-based, Primary care, Pulmonologist

## Abstract

**Background:**

In Europe, administration of an inhaled corticosteroid (ICS) combined with a long-acting β2 agonist is approved in chronic obstructive pulmonary disease (COPD) patients with a pre-bronchodilator FEV_1_ < 60% predicted normal, a history of repeated exacerbations, and who have significant symptoms despite regular bronchodilator therapy. Minimal data are available on the use of the fluticasone propionate/salmeterol xinafoate combination (FSC) in the real-life COPD setting and prescription compliance with the licensed specifications.

**Methods:**

A French observational study was performed to describe the COPD population prescribed with FSC, prescription modalities, and the coherence of prescription practices with the market authorized population. Data were collected for patients initiating FSC treatment (500 μg fluticasone propionate, 50 μg salmeterol, dry powder inhaler) prescribed by a general practitioner (GP) or a pulmonologist, using physician and patient questionnaires.

**Results:**

A total of 710 patients were included, 352 by GPs and 358 by pulmonologists. Mean age was over 60 years, and 70% of patients were male. More than half were retired, and overweight or obese. Approximately half were current smokers and one-third had cardiovascular comorbidities. According to both physician evaluation and GOLD 2006 classification, the majority of patients (>75%) had moderate to very severe COPD. Strict compliance by prescribing physicians with the market-approved population for dry powder inhaler SFC in COPD was low, notably in ICS-naïve patients; all three conditions were fulfilled in less than a quarter of patients with prior ICS and less than 7% of ICS-naïve patients.

**Conclusions:**

Prescription of dry powder inhaler SFC by GPs and pulmonologists has very low conformity with the three conditions defining the licensed COPD population. Prescription practices need to be improved and systematic FEV_1_ evaluation for COPD diagnosis and treatment management should be emphasized.

## Background

The economic and social burden of chronic obstructive pulmonary disease (COPD) is considerable, with a projection that COPD will be the third leading cause of death by 2020 [1]. Pharmacologic management for patients suffering from COPD is based on the use of bronchodilators, associated or not with a corticosteroid [2]. The therapy prescribed depends on COPD severity, prior treatment(s), their efficacy and tolerance, and patient preference regarding inhalers. In the most recent GOLD (Global initiative on Obstructive Lung Disease) global strategy document, four patient categories are defined (A, B, C and D) based on exacerbation risk and symptoms burden [2]. Exacerbation risk is assessed according to GOLD category for forced expiratory volume in 1 second (FEV_1_) and past history of exacerbations, while symptoms burden is assessed by the modified Medical Research Council (mMRC) dyspnea grading scale and/or the COPD Assessment Test (CAT), based on the worst case scenario. For patients with FEV_1_ < 50% predicted normal or a history of at least two exacerbations per year or one hospitalization for COPD exacerbation or both, combinations of inhaled corticosteroids (ICS) and long-acting β2 agonists (LABA) are recommend among first-line treatments.

Administration of the ICS fluticasone propionate with the LABA salmeterol xinafoate (fluticasone /salmeterol combination, FSC) with a dry powder inhaler was approved in Europe in 2003, at a dose of 500 μg fluticasone propionate with 50 μg salmeterol twice daily for treatment of COPD patients with a pre-bronchodilator FEV_1_ < 60% predicted normal, a history of repeated exacerbations, and who have significant symptoms despite regular bronchodilator therapy. The change of the FEV_1_ threshold from <50% in the GOLD guidelines [2] to <60% was mainly influenced by the TORCH study [3].

Coherence between published COPD guidelines and clinical prescription practices in the real-world setting has been brought into question [4-6]. Several reports highlight that a high proportion of patients who are prescribed inhaled medications, have not undergone spirometric testing to confirm diagnosis and severity [7-11]. Data on FSC use in the real-life COPD setting are scarce and co-therapies in patients prescribed with this combination as long-term treatment have not been reported.

Following a request by the French Health Authorities, a national, prospective, population-based, observational study was performed in adult COPD patients initiating treatment with fluticasone propionate/salmeterol (500/50 μg) administered with a dry powder inhaler, prescribed by a general practitioner (GP) or pulmonologist. The study purpose was twofold; the first step was to collect data describing patient characteristics and treatment modalities in this population, and to analyze the coherence of the prescription population with the licensed COPD population according to the Summary of Product Characteristics (SPC). In the second step, clinical outcomes after 12-months treatment with SFC in this population were evaluated. The present article reports results regarding the first of these two goals.

## Methods

### Study design

This was a prospective observational cohort study with 1-year follow-up in COPD patients initiating treatment with FSC delivered via a dry powder inhaler (Seretide® Diskus®, 500/50 μg). It was performed in France between March 2008 and July 2009. Patients were included by GPs and pulmonologists who were randomly selected from a validated national database of all registered physicians (Cegedim). GPs agreeing to participate were requested to include two to four consecutive eligible patients and pulmonologists to include six to nine consecutive patients. Data were collected at inclusion and at routine follow-up visits using physician questionnaires and patient self-administered questionnaires. The study was approved by the French Advisory Committee for Data Processing in Health Research (CCTIRS) and the French Data Protection Authority (CNIL) and was conducted in accordance with the Declaration of Helsinki and local regulations (etrack number: 108314). Signed informed consent was obtained from all patients.

### Patient population

To be eligible, patients had to be clinically diagnosed with COPD, initiating FSC therapy prescribed by a GP or a pulmonologist at inclusion, aged 40 years or older, and be current or former smokers with a history of at least 15 pack-years. Patients were ineligible if they had asthma, tuberculosis, cystic fibrosis, any other pulmonary condition, or cancer at inclusion, had received anticancer treatment during the previous 3 years, or were participating in another clinical or epidemiologic study.

### Questionnaires and data collection

Patient data reported by the physician at inclusion included sociodemographics, disease history (including smoking) and the following cardiovascular comorbidities: (i) cardiovascular diseases including heart failure, coronary heart disease, peripheral vascular disease, and other cardiovascular diseases (e.g., arrhythmias, valve diseases) but excluding metabolic risk factors (diabetes, hypercholesterolemia); (ii) hypertension. In addition, spirometric measurements, blood gas levels and weight were recorded at inclusion and, when performed, at each follow-up visit. Cough, expectoration, and wheezing during the 3 months prior to inclusion or any follow-up visits were reported, as well as dyspnea (modified MRC scale). Other variables included exacerbations and their management during the 12 months prior to inclusion or between visits, along with COPD treatments (bronchodilators, corticosteroids, antibiotics, physician and hospital visits, vaccinations, non-drug therapies) over the same period. On the day of enrollment, a patient self-administered questionnaire was used to evaluate dyspnea (Borg scale [12]) and quality of life (QoL; Clinical COPD Questionnaire, CCQ) [13], on the basis of the week prior to the visit. Demographics and practice data were collected for physicians.

### Evaluations and statistical analyses

A minimum of 267 patients per group (i.e., included by GPs or by pulmonologists) was required to estimate the frequency of any event occurring in about 50% of patients with a 5% type I error and a precision (i.e. maximum variation allowed around the estimate) of 6%. COPD severity was classified according to GOLD 2006 [14], taking into account FEV_1_, and to GOLD 2011 (which was not available at the time the study was performed) [15] taking into account FEV_1_, dyspnea MRC score, and history of exacerbations. Prescriptions were considered compliant with the SPC when the following three conditions were fulfilled at inclusion: 1) FEV_1_ < 60% predicted; 2) a history of repeated exacerbations (at least two over the last year, defined as an emergency department visit, hospitalization, or a course of oral corticosteroids or antibiotics for respiratory problems); 3) significant symptoms despite regular bronchodilator therapy alone (with a long-acting β2-mimetic or long-acting anticholinergic agent).

Analyses were performed according to physician specialty using descriptive statistics. Data were further analyzed in terms of prior ICS intake versus ICS-naïve. A conservative approach was used such that missing data for FEV_1_, exacerbations and long-acting bronchodilators criteria were considered as non-fulfillment of the SPC criteria. A first sensitivity analysis was performed excluding patients without FEV_1_ data given the high proportion of patients for whom this examination was not performed, and a second analysis excluded prior repeated exacerbations, given that this is a composite criterion and considered to be less reliable than other criteria.

Agreement between investigator-assessed and GOLD 2006 classifications was evaluated with a weighted Kappa test. Comparisons within GP or pulmonologist populations (ICS-naïve versus ICS-treated patients; including versus non-including physicians) were performed using student’s t-tests and variance analysis (after a Levene test for homogeneity of variance) for quantitative data. Normality was confirmed with histograms and Shapiro-Wilk tests. Satterthwaite’s approximation or Kruskal-Wallis tests were used in cases of unequal variance. Non-parametric tests were applied in the absence of normal distribution. A Wilcoxon test was used for paired series. Chi^2^ or Fisher exact tests were used to compare qualitative data. Significance comparisons of GP versus pulmonologist patient populations were not performed given that these populations were recruited in different settings during partially different time periods. A significance threshold of 0.05 was used and analyses were performed with SAS (v.9.1, SAS Institute, North Carolina USA).

## Results

**Figure 1:**
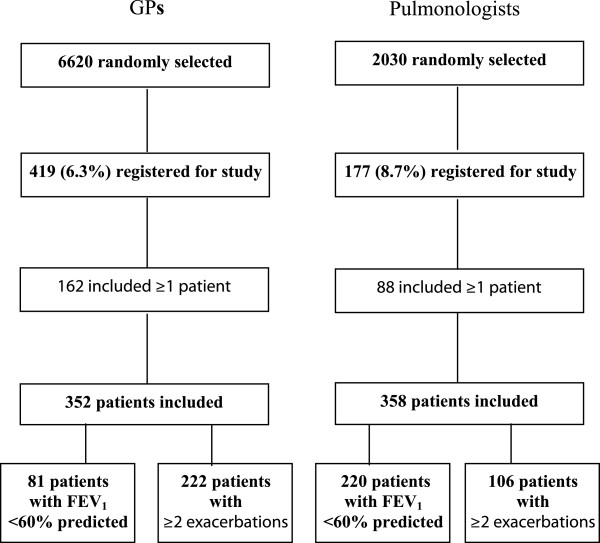
Flow diagram of physician and patient populations.

Overall 6.3% of the GPs (419 of 6620) and 8.7% of pulmonologists (177 of 2030) who were contacted agreed to participate (Figure 1). Of them, 162 GPs and 88 pulmonologists included at least one patient, with GPs including a median of two patients while pulmonologists included a median of 4.5 patients. A comparison of demographic and practice characteristics of physicians including patients with those of the national population of physicians and also with physicians who agreed to participate but did not include patients are provided in Additional file 1.

### Patient population

A total of 710 patients initiating an SFC prescription were included, 352 by GPs and 358 by pulmonologists (Figure 1). Sociodemographic and clinical characteristics are presented in Table 1. Mean patient age was over 60 years and more than half of the included patients were retired (61%) and overweight or obese (57%). Almost half the patients were current smokers and cardiovascular comorbidities were reported in approximately one-third of the population. Some differences in characteristics were apparent between the GP and pulmonologist populations, with the latter having a higher proportion of patients who were retired, male, had hypertension or coronary heart disease, while fewer pulmonologist patients were current smokers. See Additional file 2 for a description of differences in patient characteristics between patients participating versus those who did not.

**Table 1:** Main sociodemographic characteristics at inclusion and clinical profile of COPD patients prescribed with FSC

	**GPs**	**N patients (n = 352)**	**Pulmonologists**	**N patients (n = 358)**
**Age in years, mean (SD)**	61.6 (11.4)	352	65.3 (11.5)	358
**Male, N (%)**	227 (64.5%)	352	269 (75.1%)	358
**Professional status, N (%)**		352		357
Working	122 (34.7%)		57 (16.0%)	
Retired	186 (52.8%)		246 (68.9%)	
Other	44 (12.5%)		54 (15.1%)	
**BMI (kg/m**^**2**^**), N (%)**		349		358
Underweight (<18)	10 (2.9%)		25 (7.0%)	
Normal (18–25)	131 (37.5%)		138 (38.5%)	
Overweight (25–30)	133 (38.1%)		121 (33.8%)	
Obese (≥ 30)	75 (21.5%)		74 (20.7%)	
**Current smoker, N (%)**	195 (55.4%)	352	127 (35.6%)	357
**Pulmonary hypertension, N (%)**	12 (3.5%)	343	32 (9.2%)	348
**≥1 cardiovascular comorbidity, N (%)**	116 (33.0%)	352	138 (38.5%)	358
Heart failure	44 (12.5%)		43 (12.0%)	
Coronary heart disease	35 (9.9%)		62 (17.3%)	
Peripheral artery disease	48 (13.6%)		45 (12.6%)	
Other^1^	35 (9.9%)		55 (15.4%)	
**COPD severity (investigator), N (%)**		352		358
Mild	49 (13.9%)		14 (3.9%)	
Moderate	197 (56.0%)		151 (42.2%)	
Severe	100 (28.4%)		152 (42.4%)	
Very severe	6 (1.7%)		41 (11.5%)	
**COPD severity (GOLD 2006), N (%)**		137		323
Grade 1 (mild)	31 (22.6%)		16 (5.0%)	
Grade 2 (moderate)	62 (45.3%)		183 (56.6%)	
Grade 3 (severe)	13 (9.5%)		38 (11.8%)	
Grade 4 (very severe)	31 (22.6%)		86 (26.6%)	
**COPD severity (GOLD 2011), N (%)**^**2**^		136		319
Group A: Low risk, less symptoms	20 (14.7%)		71 (22.3%)	
Group B: Low risk, more symptoms	15 (11.0%)		72 (22.6%)	
Group C: High risk, less symptoms	45 (33.1%)		52 (16.3%)	
Group D: High risk, more symptoms	56 (41.2%)		124 (38.9%)	
**Median time in years since diagnosis (range)**	5.0 (0–40)	347	4.0 (0–30)	352
**FEV**_**1**_**, mean (SD)**^**3**^				
% predicted	60.5 (22.8)	145	53.6 (16.7)	334
**Arterial blood gas (mmHg), mean (SD)**				
PaO_2_	72.4 (12.8)	79	70.0 (11.7)	221
PaCO_2_	42.1 (6.2)	66	41.2 (6.0)	218
**Dyspnea (MRC grade), N (%)**		349		353
1 (strenuous exercise)	58 (16.6%)		24 (6.8%)	
2 (walking upstairs or uphill)	146 (41.8%)		114 (32.3%)	
3 (walking on the flat)	96 (27.5%)		103 (29.2%)	
4 (walking slowly)	35 (10.0%)		77 (21.8%)	
5 (daily activities)	14 (4.0%)		35 (9.9%)	
**Chronic symptoms, N (%)**^**4**^				
Daily expectorations	261 (74.6%)	350	232 (65.4%)	355
Daily cough	307 (87.7%)	350	287 (81.1%)	354
Daily expectorations + cough	245 (70.0%)	350	224 (63.3%)	354
**Quality of life (CCQ) score, median**^**5**^		304		336
Symptoms	3.3		2.8	
Functional status	2.5		2.3	
Mental status	2.0		2.0	
Total	2.7		2.4	
**History of repeated exacerbations, N (%)**^**6**^	222 (63.1%)	352	106 (29.6%)	358

^1^Excluding vascular (hypertension) and metabolic conditions (diabetes, hypercholesterolemia).

^2^A: GOLD 1–2 and < 1 exacerbation/year, MRC < 2; B: GOLD 1–2 and < 1 exacerbation/year, MRC ≥ 2; C: GOLD 3–4 and/or ≥ 2 exacerbations/year, MRC < 2; D: GOLD 3–4 and/or ≥ 2 exacerbations/year, MRC ≥ 2 [15].

^3^An additional 8 GP patients had FEV_1_ results which were aberrant and were excluded from the analysis; for one pulmonologist patient only FEV_1_ (L) was available (% FEV_1_ was missing).

^4^During the 3 months prior to initiation.

^5^During 7 days prior to initiation; score on a scale of 0 to 6 where 6 is the worst score.

^6^During the 12 months prior to initiation; exacerbation was defined as at least two of the following separated by at least 7 days over the last year: an emergency department visit, hospitalization, or a course of oral corticosteroids or antibiotics for respiratory problems.

### COPD profile and therapeutic management

At inclusion, the majority of patients had moderate to very severe COPD according to both the physician’s judgment and the GOLD-defined severity of airflow obstruction [14] (Table 1). A higher proportion of patients had very severe disease according to the GOLD criteria versus investigator judgment, which was confirmed by Kappa evaluations showing that agreement between the physician and the GOLD evaluations was poor for GPs (Kappa = 0.25) and moderate for pulmonologists (Kappa = 0.52). According to GOLD 2006 classification, pulmonologists included a higher proportion of patients with severe to very severe disease relative to GPs. FEV_1_ data were available in 335 patients included by pulmonologists (94%), but only 153 of the 352 patients included by GPs (43%, including 8 patients with an aberrant result considered missing in the analysis). Pulmonologist patients had a poorer mean FEV_1_% predicted and worse dyspnea than patients in the GP group. See Additional file 2 for a description of differences in baseline characteristics in GP patients with FEV_1_ available versus those without and for a comparison of investigator-assessed COPD severity versus GOLD 2006 in patients with FEV_1_ data.

Chronic symptoms were common in both groups, with over 80% having chronic cough, and approximately two-thirds of patients had both daily expectorations and cough (Table 1). QoL outcomes were mid-range and similar in the two groups, although symptom scores were poorer in the GP group. A history of repeated exacerbations was reported in 63% of GP patients and 30% of pulmonologist patients. Nonetheless, incidence of emergency department visits and hospitalizations was low overall with in most cases no more than one visit over 12 months, but were more common in the pulmonologist group than the GP group (Table 2). Patients in the GP group were more likely to be prescribed antibiotics and corticosteroids. Pneumococcal vaccinations had been administered in less than half the population and along with flu vaccines, were more common among GP patients than pulmonologist patients.

**Table 2:** Therapeutic management of COPD prior to initiating fluticasone/salmeterol

	**GPs**	**N patients (n = 352)**	**Pulmonologists**	**N patients (n = 358)**
**General care, N (%)**^**1**^				
Consultation with GP	320 (91.4%)	350	251 (73.6%)	341
Consultation with specialist	173 (49.6%)	349	215 (60.6%)	355
Emergency visits	31 (8.9%)	348	53 (14.9%)	356
Hospitalizations	38 (10.9%)	350	71 (19.8%)	358
Oral corticosteroids	220 (63.0%)	349	120 (33.7%)	356
Antibiotics	289 (83.5%)	346	194 (54.6%)	355
**Vaccination, N (%)**				
Flu^1^	264 (75.4%)	350	214 (60.1%)	355
Pneumococcus (within 5 years)	168 (48.1%)	349	106 (30.1%)	352
**Prior medication, N (%)**		268^2^		239^2^
Short-acting bronchodilator	37 (13.8%)		26 (10.9%)	
Long-acting ± short-acting bronchodilator	57 (21.3%)		88 (36.9%)	
ICS + long-acting bronchodilator	87 (32.5%)		92 (38.5%)	
ICS ± short-acting bronchodilator	57 (21.3%)		23 (9.6%)	
Other ICS combination	15 (5.6%)		6 (2.5%)	
Other	15 (5.6%)		4 (1.6%)	
**Concomitant medication, N (%)**^**3**^		167^4^		258^4^
Long-acting anticholinergic ± short-acting bronchodilator	53 (31.7%)		173 (67.1%)	
Short-acting bronchodilator	81 (48.5%)		71 (27.5%)	
Long-acting β-2 adrenergic agonist	18 (10.8%)		11 (4.3%)	
Other ICS	15 (9.0%)		3 (1.2%)	

^1^During the 12 months prior to inclusion.

^2^Missing data for 84 GP and 119 pulmonologist patients.

^3^At the time of FSC initiation.

^4^Missing data for 185 GP and 100 pulmonologist patients.

Prior COPD medication intake was reported in 76% of GP patients and 67% of pulmonologist patients. Co-administration of an ICS and a short-acting or long-acting bronchodilator was the most common treatment, reported in approximately half these patients (144 GP, 54%; 115 pulmonologist, 48%). The use of short-acting and/or long-acting bronchodilators (mostly anticholinergics) without ICS was reported in 35% of GP patients and 48% of pulmonologist patients (Table 2). Approximately one-third of both patient populations had received LABA with ICS.

Among the 710 patients included, a comparison of ICS-naïve patients versus those with prior ICS showed that for both GP and pulmonologist patients the ICS-naïve patient population was older (p < 0.01), had more severe COPD (investigator-assessed; p ≤ 0.0004), had been diagnosed with COPD for longer (p < 0.0001), had worse dyspnea (MRC and Borg; p ≤ 0.03) and total QoL scores (p < 0.01), were less likely to have been vaccinated (p < 0.001), have visited a specialist (p < 0.001), have received oral corticosteroid (p < 0.0001) or antibiotics (p < 0.01). In addition, GP patients with prior ICS were less likely to be current smokers (p = 0.01), more likely to have FEV_1_ data available (p < 0.0001, although there was no difference in FEV_1_ values). Pulmonologist patients with prior ICS were more likely to be female (p < 0.01), have lower percent predicated FEV_1_ (p < 0.01) and have consulted a GP (p < 0.01). See Additional file 3 for reasons for FSC prescription and further details of COPD therapies.

### Modalities of SFC prescription and compliance with recommendations

The most common Seretide® Diskus® dose prescribed was one inhalation of 500 μg fluticasone propionate/ 50 μg salmeterol twice daily (72% of GP-included patients, 83% of pulmonologist patients). A dose of two inhalations twice daily was prescribed in 23% of GP patients and 12% of pulmonologist patients.

FSC use in COPD patients in the real-life context is summarized in Table 3 in terms of compliance with the SPC, and according to prior corticosteroid intake in all patients irrespective of whether FEV_1_ data were available. Among patients having received prior ICS, compliance with at least one of the three marketing authorization conditions for prescription was reported in over 90% of patients for both GPs and pulmonologists, however compliance with all three decreased to 16% of GP patients and 26% of pulmonologist patients. In ICS-naïve patients, these proportions decreased to 73% (GP) and 80% (pulmonologist) of patients complying with at least one condition, and less than 7% of patients complying with all three conditions. Compliance with the FEV_1_ < 60% predicted normal criteria, was considerably lower in the GP group than the pulmonologist group. Conversely, patients included by pulmonologists were less likely to have fulfilled the requirement of repeated exacerbations.

**Table 3:** **Compliance of practice patterns with FSC marketing conditions for prescription according to prior ICS intake, irrespective of FEV**_**1 **_**availability**

	**Patients with prior ICS**		**ICS-naïve patients**	
	**GPs (n = 159)**	**Pulmonologists (n = 120)**	**GPs (n = 193)**	**Pulmonologists (n = 238)**
**Approved prescription condition respected, N (%)**				
FEV_1_ < 60% predicted^1^	53 (33.3%)	76 (63.3%)	28 (14.5%)	144 (60.5%)
History of repeated exacerbations	109 (68.6%)	51 (42.5%)	113 (58.5%)	55 (23.1%)
Continuous bronchodilator therapy	101 (63.5%)	97 (80.8%)	57 (29.5%)	89 (37.4%)
All conditions respected (regulatory approval criteria)	25 (15.7%)	31 (25.8%)	6 (3.1%)	16 (6.7%)
**Details of conditions respected, N (%)**				
None	15 (9.4%)	6 (5.0%)	53 (27.5%)	47 (19.7%)
FEV_1_ only	5 (3.1%)	5 (4.2%)	9 (4.7%)	69 (29.0%)
Repeated exacerbations only	27 (17.0%)	2 (1.7%)	66 (34.2%)	13 (5.5%)
Continuous bronchodilator only	18 (11.3%)	28 (23.3%)	13 (6.7%)	28 (11.8%)
FEV_1_ and repeated exacerbations only	11 (6.9%)	10 (8.3%)	8 (4.1%)	20 (8.4%)
FEV_1_ and bronchodilators only	12 (7.5%)	30 (25.0%)	5 (2.6%)	39 (16.4%)
Repeated exacerbations and bronchodilators only	46 (28.9%)	8 (6.7%)	33 (17.1%)	6 (2.5%)
FEV_1_ and repeated exacerbations +/− bronchodilators	36 (22.6%)	41 (34.2%)	14 (7.3%)	36 (15.1%)
FEV_1_ or repeated exacerbations	126 (79.2%)	86 (71.7%)	127 (65.8%)	163 (68.5%)
**N conditions respected, N (%)**				
At least one	144 (90.6%)	114 (95.0%)	140 (72.5%)	191 (80.3%)
At least two	94 (59.1%)	79 (65.8%)	52 (26.9%)	81 (34.0%)

^1^Missing FEV_1_ data were considered non-respect of condition.

## Discussion

Knowledge of real-life use of ICS/LABA combinations in the COPD setting allows for evaluation of the conformity of current prescription practices with the licensed COPD population. This can in turn be used to encourage appropriate changes in current practices to improve standards of patient care. Limited data on the use of FSC in the real-life setting have been published since the addition of COPD to the original marketing authorization for this combination based on three double-blind randomized placebo-controlled studies [16-18].

From the data obtained in this observational study of a COPD population initiating treatment with FSC administered via a dry powder inhaler, the very low rates of strict compliance with the three SPC criteria for prescription, notably in ICS-naïve patients, clearly reveal that prescribing French physicians do not adequately respect the licensing conditions. Less than a quarter of the patients with prior ICS and less than 7% of ICS-naïve patients fulfilled all three conditions. Furthermore, a small proportion of patients did not fulfill any of the conditions. When considering both two or three of the conditions, conformance improves slightly, but is still low, being reported in approximately two-thirds of patients with prior ICS, and one-third of ICS-naïve patients.

While conformance was higher for pulmonologists than GPs in terms of the number of conditions respected and for the FEV_1_ and previous bronchodilator criteria, overall rates were low for both medical specialties. In the case of GPs, the discrepancy between the licensed and real-life COPD populations is due at least in part to the high rate of missing spirometry measurements, with FEV_1_ data available for less than half of these patients. This is coherent with several studies worldwide reporting that only around a third to a half of patients undergo spirometry testing for newly diagnosed COPD or subsequently during follow-up [7,10,11,19,20]. The large proportion of missing data for FEV_1_ among GP patients limits the assessment of concordance between prescription practices and marketing authorizations and guidelines. The conservative approach used with missing data (i.e., absence of FEV_1_ considered non-respect) may have artificially lowered the rate of conformance in the GP group. Supporting this, a sensitivity analysis including only patients for whom FEV_1_ measures were available gave similar conformance rates between GPs and pulmonologists for this criterion (Additional file 4). Of note, the profile of patients without FEV_1_ measures showed them to be generally healthier than those with measures (younger, less severe COPD and dyspnea, and better QoL; see Additional file 2).

It could also be hypothesized that perceived QoL is a particularly important determinant of treatment decisions by GPs. Indeed, health-related QoL as measured by the CCQ was similar in pulmonologist and GP patients despite the latter patients having a lower dyspnea grade, less pronounced airflow obstruction and less frequent hospital visits. This illustrates that QoL measures, even when specifically designed for COPD patients, capture the impact of components other than those directly related to COPD. Interestingly, GP patients were younger and less likely to be retired than those treated by pulmonologists. Thus, it could be hypothesized that their perception of the disease’s impact is enhanced by their activity requirements, although this possibility remains to be tested. It was also noted that although pulmonologist patients are likely to have more severe COPD, pulmonologists tend to be more restrictive in the use of antibiotics and corticosteroids in patients treated in the community, as recommended in the French COPD guidelines. In these guidelines, antibiotics are recommended only when sputum is purulent, and corticosteroids are to be prescribed only for patients with severe baseline airflow obstruction and/or lack of improvement following treatment with antibiotics, if required, and bronchodilators.

Another potential reason for discrepancy between the licensed and real-life COPD populations may lie with the possibility that exacerbations were underestimated in this study since they were only assessed from patient recall; interestingly, conformance with this criterion was particularly low among pulmonologists. A low level of overall conformity was maintained in a sensitivity analysis excluding this criterion. Difficulty evaluating prior exacerbations stems from multiple sources; not only is there an absence of consensus on the definition of COPD exacerbations [21-23], but in addition, patients tend to under-report exacerbations [24], even those that have clinical significance [25].

Differences in conformity were seen according to prior corticosteroid intake, with patients having prior ICS generally less likely to conform to prescription recommendations relative to ICS-naïve patients. This may have been influenced by the fact that prior ICS treatment may have modified their clinical profile [26]. It may also be explained by the fact that this population was older, had more severe and longer duration COPD, with a higher incidence of oral corticosteroid and antibiotic intake.

Poor physician compliance with licensed conditions may also reflect a level of difficulty in applying recommendations and guidelines in the face of lack of clarity or simplicity, or in the context of individualized patient care. As reported by Corrado et al., recommendations can be considered inappropriate if other factors such as pulmonary hyperinflation, exercise capacity and tolerance, or comorbidities are not taken into account [27]. This may also explain the relatively high rates of incorrect dose prescription. Dialogue with physicians, along with improved awareness and education are needed in order to address these issues.

Analysis of populations and SPC conformance according to the GOLD 2011 classification reveals some differences compared to GOLD 2006 classification. Very severe airflow obstruction (GOLD grade 4) was reported in approximately one-quarter of the population while 40% belonged to the D GOLD 2011 category. In nearly a quarter of cases, GPs prescribed FSC to patients with GOLD 2006-defined mild COPD. This may be due to the fact that they (along with pulmonologists) tended to underestimate COPD severity relative to both 2006 and 2011 GOLD classifications (notably for most severe disease). Comparing the GOLD 2006 and 2011 classifications also shows that according to the latter, GPs were more likely than pulmonologists to see patients with more severe COPD (74% versus 55%, respectively), which was not the case with the 2006 guidelines (32% versus 38%, respectively). Furthermore, according to the GOLD 2011 classification, more patients have more severe disease, suggesting a higher rate of conformity with the SPC licensing conditions, and also that the guidelines may be evolving to adapt to the real-life situation.

Another potential study limitation concerns the representativeness of participating physicians. The low rate of physicians selected randomly from the national population who agreed to participate in the study (<10%) suggests a general reluctance to participate and consequently a potential selection bias. Nonetheless, participating physicians were broadly representative of national figures for their respective specialties in terms of age, gender and practice settings [28], although male physicians were over-represented relative to national figures. It could be hypothesized that participating physicians were more likely to be interested in the field of COPD and prescribe treatments more adequately than less interested physicians. As a consequence, the already very high rate of non-concordance between prescriptions and guidelines could be under-estimated. However, we have no means of testing this pessimistic hypothesis.

## Conclusions

The COPD population prescribed with FSC by both GPs and pulmonologists conforms poorly with the licensed population with less than a quarter of ICS-treated patients and 7% of ICS-naïve patients fulfilling all three marketing conditions. Prescribing clinicians need further education on the importance of following the SPC recommendations, including FEV_1_ evaluation, to ensure FSC is used only in COPD patients who are likely to benefit from it. Understanding the reasons behind clinicians’ decisions to prescribe FSC to non-conforming patients may help to resolve this issue.

## Abbreviations

CAT: COPD Assessment Test; CCQ: Clinical COPD questionnaire; COPD: Chronic obstructive pulmonary disease; FEV1: Forced expiratory volume in 1 sec; FSC: Fluticasone/salmeterol combination; GOLD: Global initiative for chronic obstructive lung disease; GP: General practitioner; ICS: Inhaled corticosteroids; LABA: Long-acting β2 agonists; mMRC: Modified Medical Research Council; QoL: Quality of life; SD: Standard deviation; SPC: Summary of Product Characteristics.

## Competing interests

Nicolas Roche has received over the past 5 years (i) fees for speaking, organising education, or consulting from Aerocrine, Almirall, Altana Pharma-Nycomed-Takeda, AstraZeneca, Boehringer Ingelheim, Chiesi, GlaxoSmithKline, MEDA, MSD-Chibret, Mundipharma, Novartis, Pfizer, Teva; (ii) research grants from Novartis, Nycomed, Boehringer Ingelheim and Pfizer.

Eric Van Ganse has received fees for: Consultancy from Danone, ALK-ABELLO, Chiesi; Expert testimony from UBS, IMS; Grants: IMS, CNAM, MSD, GSK; Travel/accommodations related to a consultancy from Boehringer-Ingelheim.

Philippe Serrier has received fees for speaking, consulting and attending conferences from Novartis, GSK, Pfizer, Boehringer Ingelheim, Astra Zeneca, Mundipharma, Vivisol, and Chiesi.

Bruno Housset has received fees from Aerocrine, Astra Zeneca, Boehringer Ingelheim, Chiesi, GlaxoSmithKline, Novartis, Nycomed, and Pfizer for performing continuing medical education talks and consulting work.

Déborah Poirier, Nathalie Texier and Stéphane Schück were funded by GSK to perform study analyses.

Céline Pribil and Isabelle Boucot are employees of GSK.

## Authors’ contributions

All authors approved the original study protocol, data collection and analysis plans, and the study report, as well a revising the manuscript, and have approved the submitted version. In addition, NR participated in drafting the manuscript, and NT, DP, SS performed data management and statistical analyses.

## Pre-publication history

The pre-publication history for this paper can be accessed here:

http://www.biomedcentral.com/1471-2466/14/56/prepub

## Supplementary Material

Additional file 1 Physician demographics and practice characteristics. Click here for file

Additional file 2 Comparison of patient characteristics between various groups. Click here for file

Additional file 3 COPD therapeutic management. Click here for file

Additional file 4 **Compliance in patients with available FEV**_**1**_** data.** Click here for file
