# Supplementary material for: Real-life use of fluticasone propionate/salmeterol in patients with chronic obstructive pulmonary disease: a French observational study
Source: BMC Pulm Med. 2014 Apr 2;14:56. doi: 10.1186/1471-2466-14-56 (PMC3997842; doi:10.1186/1471-2466-14-56)
Supplement: Additional file 1 — Physician demographics and practice characteristics. [file 1471-2466-14-56-S1.docx]

### Additional File 1 (pdf): Physician demographics and practice characteristics

Physicians including patients were generally middle-aged and more likely to be male. These characteristics, along with the geographic distribution of practice reflected those of the national populations of the two medical specialties (Additional Table 1) [1], although a slightly higher proportion of males was seen relative to national figures (GPs: 78% versus 61%; pulmonologists: 81% versus 67%; p<0.001).

GPs including patients were similar to pulmonologists including patients in terms of distributions of age, gender, and geographic region of practice (Additional Table 1). Pulmonologists including patients were more commonly practicing in hospitals than GPs including patients (46% versus 3%; Additional Table 2), and were less likely to be located in small towns (9% versus 56% in towns with <20,000 inhabitants).

The modalities of practice of physicians including patients were similar to those of physicians who agreed to participated but did not include patients (Additional Table 2), although pulmonologists who included patients were more likely to practice in a hospital than pulmonologists who did not (46% versus 34%) and to work independently rather than as part of a network (47% versus 33%), while GPs including patients were more likely to be from small towns (<5,000 inhabitants) than GPs who did not (35% versus 24%).

**Additional Table 1: Demographic characteristics of physicians including at least one patient compared with the national populations [1]**

|  | **GPs** | | **Pulmonologists** | |
| --- | --- | --- | --- | --- |
|  | **Including**  (n=162) | **France**  (n=101,381) | **Including**  (n=88) | **France**  (n=2,747) |
| **Age in years, N (%)** |  |  |  |  |
| < 40 | 15 (12.1%) | 17,328 (17.1%) | 10 (13.7%) | 497 (18.1%) |
| 40-49 | 46 (37.1%) | 32,505 (32.0%) | 25 (34.2%) | 832 (30.3%) |
| 50-59 | 49 (39.5%) | 39,710 (39.2%) | 30 (41.1%) | 1,110 (40.4%) |
| ≥ 60 | 14 (11.3%) | 11,838 (11.7%) | 8 (11.0%) | 308 (11.2%) |
| Missing | 38 | 0 | 15 | 0 |
| **Male, N (%)** * | 126 (77.8%) | 61,538 (60.7%) | 71 (80.7%) | 1,838 (66.9%) |
| **Region, N (%)** |  |  |  |  |
| Paris | 20 (12.3%) | 20,355 (20.1%) | 12 (13.6%) | 565 (20.6%) |
| Greater Paris | 31 (19.1%) | 15,269 (15.1%) | 13 (14.8%) | 411 (15.0%) |
| North | 15 (9.3%) | 6,623 (6.5%) | 6 (6.8%) | 178 (6.5%) |
| East | 13 (8.0%) | 8,549 (8.4%) | 7 (8.0%) | 241 (8.8%) |
| West | 25 (15.4%) | 12,719 (12.5%) | 13 (14.8%) | 322 (11.7%) |
| South-west | 19 (11.7%) | 11,623 (11.5%) | 5 (5.7%) | 292 (10.6%) |
| Central-east | 13 (8.0%) | 11,995 (11.8%) | 9 (10.2%) | 301 (11.0%) |
| Mediterranean | 26 (16.0%) | 14,248 (14.1%) | 23 (26.1%) | 436 (15.9%) |

*p < 0.001

**Additional Table 2: Modalities of practice of physicians who agreed to participate, comparing those who did versus those who did not include patients**

|  | **GPs** | | **Pulmonologists** | |
| --- | --- | --- | --- | --- |
|  | **Including patients**  (n=162) | **Not including patients**  (n=257) | **Including patients**  (n=88) | **Not including patients**  (n=89) |
| **Practice type, N (%)** |  |  |  |  |
| Private practice only | 137 (86.2%) | 219 (86.9%) | 24 (27.3%) | 30 (34.9%) |
| Private + part-time | 17 (10.7%) | 26 (10.3%) | 24 (27.3%) | 27 (31.4%) |
| Hospital-based | 5 (3.1%) | 7 (2.8%) | 40 (45.5%) | 29 (33.7%) |
| Missing | 3 | 5 | 0 | 3 |
| **Practice conditions, N (%)** |  |  |  |  |
| Group practice | 73 (49.3%) | 92 (52.6%) | 27 (52.9%) | 29 (67.4%) |
| Independent | 75 (50.7%) | 83 (47.4%) | 24 (47.1%) | 14 (32.6%) |
| Missing | 14 | 82 | 37 | 46 |
| **Practice locality, N (%)** |  |  |  |  |
| < 5,000 inhabitants | 54 (35.3%) | 45 (24.3%) | 0 (0%) | 2 (2.8%) |
| 5,000 – 20,000 inhabitants | 31 (20.3%) | 42 (22.7%) | 7 (9.2%) | 5 (7.0%) |
| 20,000 – 100,000 inhabitants | 37 (24.2%) | 39 (21.1%) | 38 (50.0%) | 32 (45.1%) |
| > 100,000 inhabitants | 18 (11.8%) | 39 (21.1%) | 23 (30.3%) | 26 (36.6%) |
| Paris region | 13 (8.5%) | 20 (10.8%) | 8 (10.5%) | 6 (8.5%) |
| Missing | 9 | 72 | 12 | 18 |

### Reference

1. DREES (French Ministry of Health Directorate for Research, Evaluation and Statistics): [*Physicians: Estimations on January 1, 2008. Statistical Series, No. 127, October 2008*] in French. http://www.drees.sante.gouv.fr/IMG/pdf/seriestat127.pdf. (Accessed September 2013)
