# Supplementary material for: Real-life use of fluticasone propionate/salmeterol in patients with chronic obstructive pulmonary disease: a French observational study
Source: BMC Pulm Med. 2014 Apr 2;14:56. doi: 10.1186/1471-2466-14-56 (PMC3997842; doi:10.1186/1471-2466-14-56)
Supplement: Additional file 2 — Comparison of patient characteristics between various groups. [file 1471-2466-14-56-S2.docx]

### Additional File 2 (pdf): Comparison of patient characteristics between various groups

**Representativeness of participating patients**

A register was kept of eligible COPD patients treated by participating GPs but who were not included in the study. A total of 440 eligible COPD patients treated by the participating GPs and 98 treated by participating pulmonologists were not included, due to refusal of consent (172 patients), target number of patients being reached (63 patients), asthma (57 patients) or another comorbidity. Non-participating patients had similar age, gender and FEV_1_ outcome as the 710 included patients. However non-participating patients were more likely to be current smokers than participating patients (GPs: 80% versus 55%; pulmonologists: 94% versus 36% respectively), and to have had COPD for longer (GPs: median 8 years versus 5 years; pulmonologists: median 10 years versus 4 years).

**Patients with and without FEV_1_ data**

Among the 710 patients included, FEV_1_ data were missing for 199 of the 352 GP patients (57%) and 23 of the 358 pulmonologist patients (6%). T he characteristics of GP patients with FEV_1_ data were compared to those without. Patients without FEV_1_ were more likely to be younger, professionally active, current smokers, have less severe COPD (investigator-assessed) and dyspnea (MRC and Borg), better QoL, to be vaccinated, and were less likely to have been vaccinated (flu or pneumococcus), have visited a specialist, have a cardiovascular comorbidity or have had an emergency visit or hospitalization during the previous year.

**Comparison of physician analysis of COPD severity versus GOLD 2006 severity in patients with FEV_1_ data**

Compared to GOLD-defined severity of airflow obstruction, GPs underestimated the proportions of patients with mild and very severe COPD while overestimating the proportion of severe cases. Pulmonologists tended to underestimate the proportion of patients with moderate and very severe COPD, although to a lesser extent than GPs, qualifying a large proportion of these patients as severe.

|  | **GP** | | **Pulmonologist** | |
| --- | --- | --- | --- | --- |
|  | **Physician opinion**  **(N=153)** | **GOLD 2006**  **(N=137*)** | **Physician opinion**  **(N=335)** | **GOLD 2006**  **(N=323*)** |
| Mild | 13 (8.5%) | 31 (22.6%) | 14 (4.2%) | 16 (5.0%) |
| Moderate | 68 (44.4%) | 62 (45.3%) | 138 (41.2%) | 183 (56.7%) |
| Severe | 70 (45.8%) | 13 (9.5%) | 143 (42.7%) | 38 (11.8%) |
| Very severe | 2 (1.3%) | 31 (22.6%) | 40 (11.9%) | 86 (26.6%) |

* Missing data for 16 GP patients and 12 pulmonologist patients
