# Supplementary material for: Real-life use of fluticasone propionate/salmeterol in patients with chronic obstructive pulmonary disease: a French observational study
Source: BMC Pulm Med. 2014 Apr 2;14:56. doi: 10.1186/1471-2466-14-56 (PMC3997842; doi:10.1186/1471-2466-14-56)
Supplement: Additional file 3 — COPD therapeutic management. [file 1471-2466-14-56-S3.docx]

### Additional File 3 (pdf): COPD therapeutic management

In almost all cases (over 93%), FSC therapy was prescribed to relieve COPD symptoms, while ease of use for the patient (52% GPs, 30% of pulmonologists), and assisting patient compliance (40% GPs, and 16% pulmonologists) were also common reasons cited.

Non-pharmacologic therapies were less frequently implemented than medications, reported in 21% of patients in the GP group and 36% of pulmonologist patients (Additional Table 3). A different profile was apparent between the GP and pulmonologist groups, with chest physiotherapy common in both, while oxygen therapy was more common with pulmonologists.

**Additional Table 3: Other therapeutic interventions for COPD prior to initiating fluticasone/salmeterol**

|  | **GPs** | **N patients (n=352)** | **Pulmonologists** | **N patients (n=358)** |
| --- | --- | --- | --- | --- |
| **Other interventions, N (%)^1^** |  |  |  |  |
| Chest physiotherapy | 41 (11.6%) | 352 | 44 (12.3%) | 358 |
| Long-term oxygen therapy | 21 (6.0%) | 352 | 54 (15.1%) | 358 |
| Pulmonary rehabilitation | 16 (4.5%) | 352 | 16 (4.5%) | 358 |
| Nocturnal ventilation | 13 (3.7%) | 352 | 21 (5.9%) | 358 |

1. Ongoing or prescribed at inclusion
