# Supplementary material for: Real-life use of fluticasone propionate/salmeterol in patients with chronic obstructive pulmonary disease: a French observational study
Source: BMC Pulm Med. 2014 Apr 2;14:56. doi: 10.1186/1471-2466-14-56 (PMC3997842; doi:10.1186/1471-2466-14-56)
Supplement: Additional file 4 — Compliance in patients with available FEV 1 data. [file 1471-2466-14-56-S4.docx]

### Additional File 4 (pdf): Compliance in patients with available FEV_1_ data

**Additional Table 4: Compliance of practice patterns with FSC marketing conditions for prescription according to prior ICS intake, in patients with FEV_1_ data**

|  | **Patients with prior ICS** | | **ICS-naive** | |
| --- | --- | --- | --- | --- |
| **Compliance, N (%)** | **GPs**  **(N=82)** | **Pulmonologists**  **(N=112)** | **GPs**  **(N=63)** | **Pulmonologists**  **(N=222)** |
| **Approved prescription condition respected** |  |  |  |  |
| FEV_1_ < 60% predicted | 53 (64.6%) | 76 (67.9%) | 28 (44.4%) | 144 (64.9%) |
| History of repeated exacerbations | 55 (67.1%) | 49 (43.8%) | 35 (55.6%) | 50 (22.5%) |
| Continuous bronchodilator therapy | 58 (70.7%) | 91 (81.3%) | 29 (46.0%) | 85 (38.3%) |
| **N conditions respected** |  |  |  |  |
| At least one | 79 (96.3%) | 108 (96.4%) | 55 (87.3%) | 183 (82.4%) |
| At least two | 62 (75.6%) | 77 (68.8%) | 31 (49.2%) | 80 (36.0%) |
| All three | 25 (30.5%) | 31 (27.7%) | 6 (9.5%) | 16 (7.2%) |
| **Combinations of conditions respected** |  |  |  |  |
| None | 3 (3.7%) | 4 (3.6%) | 8 (12.7%) | 39 (17.6%) |
| FEV_1_ only | 5 (6.1%) | 5 (4.5%) | 9 (14.3%) | 69 (31.1%) |
| Repeated exacerbations only | 5 (6.1%) | 2 (1.8%) | 9 (14.3%) | 9 (4.1%) |
| Continuous bronchodilator only | 7 (8.5%) | 24 (21.4%) | 6 (9.5%) | 25 (11.3%) |
| FEV_1_ and repeated exacerbations only | 11 (13.4%) | 10 (8.9%) | 8 (12.7%) | 20 (9.0%) |
| FEV_1_ and bronchodilators only | 12 (14.6%) | 30 (26.8%) | 5 (7.9%) | 39 (17.6%) |
| Repeat exacerbations and bronchodilators only | 14 (17.1%) | 6 (5.4%) | 12 (19.0%) | 5 (2.3%) |
